# Association between dietary and behavioral-based oxidative balance score and phenotypic age acceleration: a cross-sectional study of Americans

**DOI:** 10.4178/epih.e2024023

**Published:** 2024-01-18

**Authors:** Dongzhe Wu, Yulin Shen, Chaoyi Qu, Peng Huang, Xue Geng, Jianhong Zhang, Zhijian Rao, Qiangman Wei, Shijie Liu, Jiexiu Zhao

**Affiliations:** 1 Exercise Biological Center, China Institute of Sport Science, Beijing, China; 2 Department of Exercise Physiology, Beijing Sport University, Beijing, China; 3 School of Exercise and Health, Shanghai University of Sport, Shanghai, China; 4 National Institute of Sports Medicine, Beijing, China; 5 College of Physical Education, Shanghai Normal University, Shanghai, China

**Keywords:** Oxidative balance score, Phenotypic age acceleration, National Health and Nutrition Examination Survey

## Abstract

**OBJECTIVES:**

In light of the rise in the global aging population, this study investigated the potential of the oxidative balance score (OBS) as an indicator of phenotypic age acceleration (PhenoAgeAccel) to better understand and potentially slow down aging.

**METHODS:**

Utilizing data from the National Health and Nutrition Examination Survey collected between 2001 and 2010, including 13,142 United States adults (48.7% female and 51.2% male) aged 20 and above, OBS and PhenoAgeAccel were calculated. Weighted generalized linear regression models were employed to explore the associations between OBS and PhenoAgeAccel, including a sex-specific analysis.

**RESULTS:**

The OBS demonstrated significant variability across various demographic and health-related factors. There was a clear negative correlation observed between the higher OBS quartiles and PhenoAgeAccel, which presented sex-specific results: the negative association between OBS and PhenoAgeAccel was more pronounced in male than in female. An analysis using restricted cubic splines revealed no significant non-linear relationships. Interaction effects were noted solely in the context of sex and hyperlipidemia.

**CONCLUSIONS:**

A higher OBS was significantly associated with a slower aging process, as measured by lower PhenoAgeAccel. These findings underscore the importance of OBS as a biomarker in the study of aging and point to sex and hyperlipidemia as variables that may affect this association. Additional research is required to confirm these results and to investigate the biological underpinnings of this relationship.

## GRAPHICAL ABSTRACT

[Fig f3-epih-46-e2024023]

## Key Message

• The study found a significant negative correlation between the oxidative balance score (OBS) and phenotypic age acceleration (PhenoAgeAccel), with higher OBS associated with slower biological aging. This association was more pronounced in males, and significant in individuals with high cholesterol.

• OBS can serve as an effective biomarker for studying the aging process and its association with lifestyle and dietary factors.

## INTRODUCTION

Over the past few decades, researchers have gradually recognized the importance of oxidative stress and the antioxidant defense system in the aging process. Multiple studies have indicated [1-3] that as individuals age, the balance between oxidative and antioxidative systems shifts, leading to heightened oxidative stress. Oxidative stress is closely linked to the aging process, as oxidative stress levels in the body tend to increase with age, partly due to decreased antioxidant efficiency and increased oxidative damage [4]. Antioxidants play a vital role in this process, and a strengthened antioxidant defense mechanism can have an “anti-aging” effect [2], helping to protect the body from age-related functional impairments such as cognitive decline. Higher resistance to oxidative stress and a strengthened antioxidant defense are considered essential factors in extending the lifespan [5,6]. Furthermore, environmental, nutritional, and pharmaceutical strategies may offer possibilities for regulating oxidative stress and antioxidant balance, thereby correcting the aging process [7-10]. A deeper understanding of the role of oxidative stress in aging-related diseases and the evidence for antioxidant therapies will provide a valuable avenue for the development of potential interventions, promoting healthy longevity.

The oxidative balance score (OBS) is a comprehensive indicator designed to quantify the oxidative and antioxidant status within an individual’s body by considering lifestyle factors, dietary factors, and biomarkers associated with oxidative stress [11]. Numerous studies have shown a significant association between OBS and the risk of various diseases, including all-cause mortality [12], metabolic syndrome [13], end-stage renal disease, and cardiovascular diseases [14]. OBS is an important tool not only because it helps assess and understand the relationship between oxidative balance and disease risk but also because it is associated with antioxidantrich dietary patterns and inflammatory biomarkers [15]. Therefore, OBS holds practical value in disease risk assessment, disease progression monitoring, clinical interventions, and public health.

Phenotypic age acceleration (PhenoAgeAccel) is an indicator that reflects the difference between an individual’s biological age and their actual age, and it is used to quantify the rate of biological aging [16]. Recent research has shown significant associations between this indicator and various age-related diseases, including dementia [17] and cardiovascular diseases [18], as well as socioeconomic factors [19], providing essential information for understanding healthy aging and disease development. PhenoAgeAccel is calculated by considering multiple biological markers and clinical indicators, which are typically related to an individual’s health status, physiological function, and biological aging processes. This indicator not only reveals an individual’s rate of aging, but also predicts the risk of future age-related diseases, making it valuable in both clinical and public health settings. By assessing PhenoAgeAccel, researchers and clinicians can gain a more accurate understanding of an individual’s health status and degree of aging, leading to more effective prevention and treatment strategies.

In light of research exploring the important roles of oxidative stress and the antioxidant defense system in the aging process, as well as the potential significance of OBS and PhenoAgeAccel in disease risk assessment and aging research, we recognize the enormous potential of these biological indicators for elucidating the complex mechanisms of aging and developing effective intervention strategies. However, despite some significant findings, the detailed mechanisms of interactions, synergistic effects, and how they collectively influence the aging process and disease development across multiple levels and dimensions remain less clear.

Therefore, this study aimed to explore in-depth the relationship between OBS and PhenoAgeAccel and attempted to reveal the potential synergistic processes between OBS and human phenotypic biological aging. The preliminary hypothesis of this study is that a significant inverse association may exist between OBS and PhenoAgeAccel, implying the possibility of slowing down the aging process by improving oxidative balance.

## MATERIALS AND METHODS

### Study subjects and data sources

This study utilized data from the National Health and Nutrition Examination Survey (NHANES) conducted between 2001 and 2010. NHANES is a nationally representative survey conducted by the Centers for Disease Control and Prevention (CDC) in the United States. Its primary aim is to assess the health and nutritional status of both adults and children across the country. This study adheres to the guidelines of the Strengthening the Reporting of Observational Studies in Epidemiology (STROBE) statement. The survey is conducted every 2 years by the National Center for Health Statistics and the CDC, employing a multi-stage probability sampling design to examine approximately 10,000 non-institutionalized individuals from various regions in the United States. Data collection includes household interviews and physical examinations. During the interviews, participants answer questions regarding demographic, socioeconomic, dietary, and health-related variables, while the physical examinations involve measuring medical, dental, and physiological and biochemical indicators.

**Figure 1.**
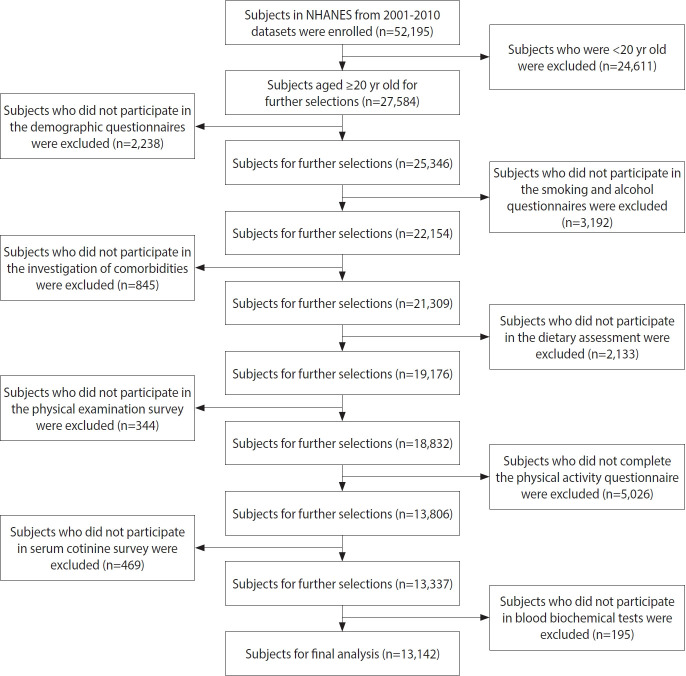
Recruitment flowchart for participants. NHANES, National Health and Nutrition Examination Survey.

For this study, after a screening process, a total of 13,142 adult participants aged 20 years and above were included in the final analysis. The screening primarily involved the exclusion of participants who did not meet the age criteria and those who did not complete relevant questionnaires and physical examinations. A complete case analysis approach was employed for data analysis. The detailed screening process is illustrated in Figure 1.

### Definition of the oxidative balance score

OBS is a comprehensive index that quantifies an individual’s oxidative and antioxidative levels by assessing their dietary and lifestyle factors [11,20,21]. This index consists of 2 major categories: pro-oxidants and antioxidants, including but not limited to total fat, iron, alcohol, dietary fiber, various vitamins, and minerals, among others. Each component is assigned scores based on sex-specific and tertiles of the components. Antioxidant components have a score ranging from 0 to 2, while pro-oxidant components have a score ranging from 2 to 0. These scores are then summed to obtain the total OBS score (Supplementary Material 1), where a higher OBS score indicates more significant antioxidant exposure, reflecting better antioxidant activity.

To accurately calculate OBS, information about individuals’ diet, physical activity, alcohol consumption, and smoking habits was collected. Dietary data were obtained through two 24-hour dietary recalls, including a face-to-face interview and a telephone interview, with the average of the 2 being used. Physical activity data were collected by asking participants whether they engaged in any moderate or vigorous physical activities, exercise, or recreational activities in the past week. This included work-related activities, leisuretime sports, and commuting activities (such as walking or cycling), quantified using metabolic equivalent (MET) scores to assess the intensity of physical activity. Additionally, serum cotinine levels were determined using the isotope dilution-high-performance liquid chromatography/atmospheric pressure chemical ionization tandem mass spectrometry method as an indicator of smoking status. The comprehensive analysis and scoring of all these data provide a scientific basis for assessing an individual’s oxidative balance status, making OBS an effective tool for accurately reflecting an individual’s oxidative balance state. Detailed information is shown in Supplementary Material 1.

### Definitions of phenotypic age acceleration

This study calculated individual phenotypic age based on previous representative research on phenotypic age [22]. Phenotypic age is calculated by considering both actual age and 9 biological markers, including albumin, creatinine, glucose, logarithmically transformed C-reactive protein (CRP), lymphocyte percentage, mean cell volume, red cell distribution width, alkaline phosphatase, and white blood cell count. It is computed through a parametric modeling of 2 Gompertz proportional hazards models, one using all 10 selected variables, and the other using only actual age. These biological markers were selected after a ten-fold cross-validation of mortality using a Cox proportional hazards elastic net model.

PhenoAgeAccel in this study is calculated by regressing phenotypic age on actual age using a linear model [16]. For example, if 2 individuals are both 50 years old, but one exhibits a younger physiological state and vitality while the other appears older due to health issues or an unhealthy lifestyle, PhenoAgeAccel measures the difference between their physiological state and their actual age. It serves as a low-optimality indicator, with lower values indicating a slower biological aging process. PhenoAgeAccel is similar to an indicator that quantifies an individual’s physiological state relative to their actual age, helping to understand the difference in physiological aging rate relative to actual age. The specific formula for PhenoAgeAccel is as follows:


$$\text{Phenotypic age}=141.50+\frac{\ln[-0.00553\times \ln(1-\mathrm{xb})]}{0.09165}\;\mathrm{xb}=-19.907-0.0336\times \mathrm{albumin}+0.0095\times \mathrm{creatinine}+0.0195\times \mathrm{glucose}+0.0954\times \ln(\mathrm{CRP})-0.0120\times \mathrm{lymphocyte percent}+0.0268\times \mathrm{mean cell volume}+0.3356\times \mathrm{red cell distribution width}+0.00188\times \mathrm{alkaline phosphatase}+0.0554\times \mathrm{white blood cell count}+0.0804\times \mathrm{chronological age}$$

### Covariates

In this study, the included covariates encompassed the following variables: age; sex (male, female); ethnicity (Mexican-American, non-Hispanic Black, non-Hispanic White, other races); educational level (less than high school, high school, higher than high school); poverty income ratio (PIR), calculated by dividing household (or individual) income by the poverty threshold for the survey year (low income PIR≤ 1.3, moderate income 1.3< PIR< 3.5, high income ≥ 3.5); marital status (married/cohabiting, married, widowed/divorced/separated); body mass index (BMI; < 25.0, 25.0-29.9, ≥ 30.0 kg/m^2^); smoking status categorized into three groups: never (smoked fewer than 100 cigarettes in a lifetime), former (smoked more than 100 cigarettes in their lifetime but currently do not smoke), current (smoked more than 100 cigarettes in their lifetime and currently smoke occasionally or daily); alcohol consumption categorized into five groups: never (drank alcohol less than 12 times in a lifetime), former (drank alcohol at least once in the past 12 years but did not drink in the past year, or did not drink in the past year but drank at least 12 times in their lifetime), light (female: ≤ 1 drink/day; male: ≤ 2 drink/day), moderate (female: ≤ 2 drink/day; male: ≤ 3 drink/day), and heavy (female: ≤ 3 drink/day; male: ≤ 4 drink/day); the diagnostic criteria for hypertension included: (1) whether a doctor or healthcare professional had diagnosed the participant with hypertension, (2) whether they used antihypertensive medication, and (3) having systolic blood pressure ≥ 140 mmHg and diastolic blood pressure ≥ 90 mmHg in three consecutive blood pressure measurements; the diagnostic criteria for hyperlipidemia included: (1) triglycerides ≥150 mg/dL, (2) serum total cholesterol ≥200 mg/dL, low-density lipoprotein ≥ 130 mg/dL, high-density lipoprotein < 40 mg/dL (in male), < 50 mg/dL (in female), and (3) the use of lipid-lowering medications; the diagnostic criteria for diabetes included: (1) whether a doctor or healthcare professional had diagnosed the participant with diabetes, (2) glycated hemoglobin ≥ 6.5 mmol/L, (3) fasting glucose ≥ 7.0 mmol/L, and (4) the use of antidiabetic medications.

### Statistical analysis

This study adhered to the complex sampling procedures of the NHANES and computed complex survey weights according to the NHANES analysis guidelines. Weighted data were utilized in the analysis to produce estimates with national representativeness. Continuous variables in this study were presented as means (standard errors), while categorical variables were expressed in actual numbers (weighted percentages). Group differences were assessed using various statistical tests, such as one-way analysis of variance for continuous variables and the chi-square test for categorical variables.

Weighted generalized linear regression models were used to determine the correlation between OBS and PhenoAgeAccel. In the crude model, only the effect of OBS on PhenoAgeAccel was considered. In model 1, other potential confounding factors were adjusted, including age, sex, race, PIR, education level, BMI, marital status, smoking status, and alcohol consumption. Building upon model 1, model 2 further adjusted for comorbidities such as diabetes, hypertension, and hyperlipidemia. Additionally, restricted cubic splines (RCS) were employed to examine the non-linear relationship between OBS and PhenoAgeAccel.

Subgroup analysis was conducted to explore the association between OBS and PhenoAgeAccel between the sexes. The covariates selected for the model followed those mentioned in the overall model above. Finally, control variables were included in the interaction effect test model to further investigate whether there were interaction effects between control variables and OBS and PhenoAgeAccel. A 2-sided p-value < 0.05 significance threshold was used to determine statistically significant differences. All analyses were conducted using R version 4.2.1 (R Foundation for Statistical Computing, Vienna, Austria).

### Ethics statement

This study was conducted in accordance with the 2000 revised version of the Helsinki Declaration, and all participants provided informed consent. The investigation adhered to the Helsinki Declaration, and the research ethics review was conducted under Protocol numbers 98-12 (2001-2004) and Continuation of Protocol 2005-06 (2005-2010). The authors are committed to ensuring accountability for all aspects of the work’s accuracy or integrity and will appropriately investigate and resolve any related issues.

## RESULTS

### Baseline characteristics of the study population

Table 1 displays the characteristics of the study population from the 2001-2010 NHANES, grouped into quartiles based on OBS. There were significant differences (p< 0.05) among the different quartile groups in terms of age, race, BMI, education level, marital status, PIR, smoking status, alcohol consumption, diabetes, hypertension, hyperlipidemia, phenotypic age, PhenoAgeAccel, albumin, creatinine, glucose, CRP, red blood cell distribution width, alkaline phosphatase, mean cell volume, and white blood cell count. However, there was no significant difference in lymphocyte percent (p> 0.05).

### Association analysis between oxidative balance score and phenotypic age acceleration in American adults

**Figure 2.**
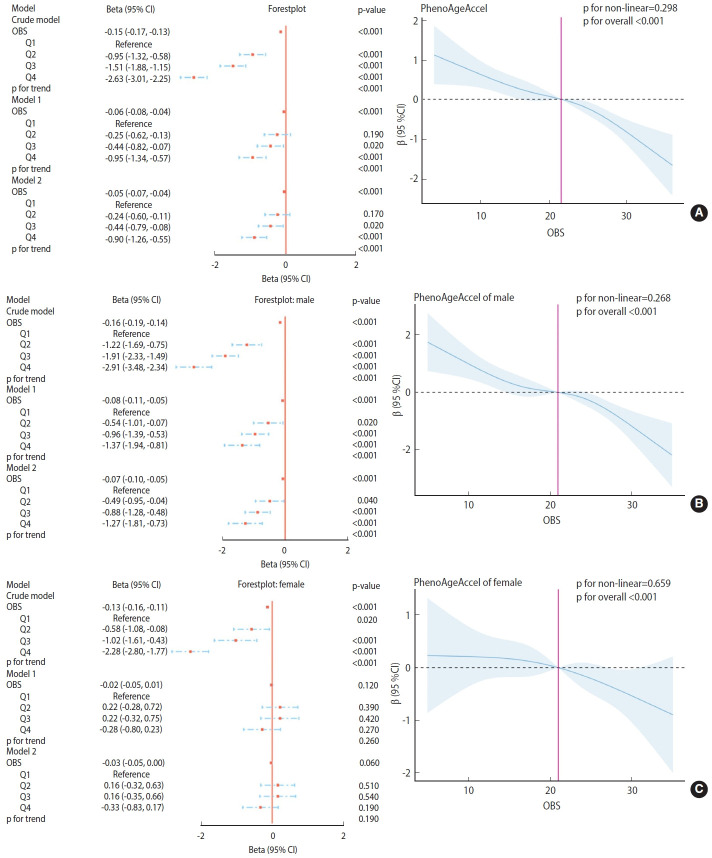
Association analysis between oxidative balance score (OBS) and phenotypic age acceleration (PhenoAgeAccel) in American adults (A) total, (B) male, and (C) female. Crude model: Unadjusted model; Model 1: Adjusted for age, sex, race, poverty to income ratio, education level, body mass index, marital status, smoke status, alcohol status; Model 2: Model 1+diabetes, hypertension and hyperlipidemia. The adjusted restricted cubic spline model illustrates the association between PhenoAgeAccel and OBSs among all participants. This model has been covariate-adjusted based on model 2. The blue solid line and the shaded blue area represent the estimated regression coefficient Beta and its 95% confidence interval (CI).

The relationship between OBS and PhenoAgeAccel was assessed using generalized linear regression weighted models, as depicted in Figure 2.

As illustrated in the models in Figure 2A, in the crude model, OBS (as a continuous value) showed a significant negative correlation with PhenoAgeAccel (p < 0.001). Compared to the lowest quartile (Q1), Q2, Q3, and Q4 of OBS demonstrated a significant negative correlation with PhenoAgeAccel (p< 0.001). This trend remained highly significant (p< 0.001) in the fully adjusted model (model 2), indicating a consistent and robust relationship. The RCS plots did not exhibit a significant non-linear trend between OBS and PhenoAgeAccel in the overall population (p for non-linearity= 0.298; p for overall < 0.001).

As shown in the models in Figure 2B, for males, OBS (as a continuous value) was significantly negatively correlated with PhenoAgeAccel in the crude model (p< 0.001). Compared to the lowest quartile (Q1), quartiles Q2, Q3, and Q4 of OBS also showed a significant negative correlation with PhenoAgeAccel (p < 0.001). This trend remained highly significant (p< 0.001) in the fully adjusted model (model 2), suggesting a consistent and robust relationship for males. The RCS plots did not demonstrate a significant non-linear trend between OBS and PhenoAgeAccel in the male subgroup (p for non-linearity= 0.268; p for overall < 0.001).

As depicted in the models in Figure 2C, for females, OBS (as a continuous value) was significantly negatively correlated with PhenoAgeAccel in the crude model (p< 0.001). Compared to the lowest quartile (Q1), quartiles Q2, Q3, and Q4 of OBS also showed a significant negative correlation with PhenoAgeAccel (p< 0.001). However, in both model 1 and the fully adjusted model (model 2), this correlation was not significant across all quartiles and as a continuous value, indicating sex-specific differences in the OBS-PhenoAgeAccel relationship. The RCS plots did not reveal a significant non-linear trend between OBS and PhenoAgeAccel in the female subgroup (p for non-linearity= 0.659; p for overall < 0.001).

### Interaction effect test

Table 2 presents the results of interaction effect tests. The selected stratification variables in this study, including age, race, education level, BMI, marital status, PIR, smoking status, alcohol consumption, hypertension, and diabetes, did not exhibit significant interaction effects on the association between OBS and PhenoAgeAccel (p for interaction > 0.05). However, sex and hyperlipidemia showed significant interaction effects on the association between OBS and PhenoAgeAccel (p for interaction < 0.05), suggesting that the impact of OBS on PhenoAgeAccel may vary depending on an individual’s sex and the presence of high cholesterol levels.

## DISCUSSION

This study, based on the 2001-2010 NHANES dataset, systematically explored the potential relationship between OBS and PhenoAgeAccel. We found that, after multivariable adjustments, there was a significant negative correlation between OBS and PhenoAgeAccel. This trend was consistent in different sex subgroups, especially more pronounced in higher OBS quartiles. However, the non-linear trend of this association was not significant, suggesting that the relationship between OBS and PhenoAgeAccel exhibits stable linear characteristics and is not influenced by specific thresholds. In the examination of interaction effects, sex and high cholesterol levels showed significant interactions with the association between OBS and PhenoAgeAccel, while other variables such as age, race, education level, etc., did not exhibit significant interaction effects. This finding indicates that sex and high cholesterol levels may play a moderating role in the relationship between OBS and PhenoAgeAccel. Overall, this significant negative correlation suggests that higher levels of OBS may be associated with a slower biological aging process. However, the specific biological mechanisms behind this and the clinical implications require further in-depth research.

In the exploration of the potential relationship between OBS and PhenoAgeAccel, remarkable research findings and challenges have been encountered. Studies by Romeu et al. [23] and Kozakiewicz and Collegues [24,25] have both revealed a close connection between oxidative stress and aging, providing valuable insights into how OBS may affect the aging process. However, they have also raised an unexpected issue—namely, that enhanced antioxidant capacity may not necessarily provide sufficient protection, indicating that when seeking strategies to delay aging, solely relying on boosting an individual’s antioxidant capacity may not be enough. Additionally, research by Poljsak & Milisav [26] has further elucidated the role of antioxidants in alleviating oxidative stress, but their effectiveness in slowing down aging is not consistent. This indicates that while antioxidants play a role in modulating OBS, relying exclusively on them might not be sufficient to significantly slow down the aging process. A more holistic approach, which encompasses a range of factors including genetic, metabolic, and environmental aspects, may be required to effectively manage the body’s oxidative balance and its impact on aging.

Furthermore, the findings by Ilori and Collegues [14,27], indicating a reverse association between OBS and the progression of end-stage renal disease, provide valuable clues, suggesting that in the context of certain chronic diseases, changes in OBS may be related to disease progression and the rate of biological aging. This corresponds to the study by Dzięgielewska-Gęsiak et al. [28], according to which insulin resistance and oxidative stress may be outcomes rather than causes of aging. The research by Cohen et al. [29] highlighted the importance of not focusing on individual markers of oxidative damage or antioxidant status in all antioxidant/oxidative stress research but rather understanding how they integrate into the oxidative balance system, as free radicals can play both beneficial and damaging roles in signaling pathways, and therefore should not be viewed in isolation.

In this series of findings, we also observed significant interactions between sex and high cholesterol levels with OBS and PhenoAgeAccel. This suggests that these factors may play a moderating role in the relationship between OBS and biological aging, providing new directions for future research. This emphasizes the need to consider the potential impact of these variables when exploring the complex relationship between OBS and biological aging comprehensively. Finally, the research of Traustadóttir et al. [30], by revealing the crucial role of fat in the biological aging process, offers a new perspective on the impact of lifestyle factors such as diet and exercise on aging. It underscores the potential significance of lifestyle adjustments in slowing down the aging process. The studies mentioned above provide further theoretical references and scientific data supporting the results of this study.

Previous OBS research has been largely associated with chronic diseases or health outcomes, but there has been relatively little research on associations with related aging, especially the study of related physiological mechanisms. This paper logically summarizes previous correlation studies to further support the potential physiological mechanisms involved in this study. Based on the components of OBS and a series of scientific studies, it is possible to explore the potential physiological mechanisms between OBS and PhenoAgeAccel. OBS takes into account various pro-oxidants, antioxidants, and related lifestyle factors, all of which are closely linked to intracellular oxidative stress and antioxidant defense mechanisms, and therefore have a direct connection to the cellular aging process. Finkel & Holbrook [31]’s research emphasizes the central role of free radicals and oxidative stress in cellular aging, which aligns with the consideration of pro-oxidants in OBS. Halliwell [32]’s work further revealed the critical role of antioxidants in neutralizing free radicals and reducing oxidative damage, helping to elucidate the importance and mechanism of antioxidants in OBS. Moderate physical activity can enhance antioxidant defenses, as confirmed by Warburton et al. [33], while Popkin et al. [34] demonstrated that poor dietary habits, especially excessive alcohol consumption, can exacerbate oxidative stress. In addition, various nutrients involved in OBS, such as fats, iron, vitamins, and minerals, are closely related to physiological processes such as cellular energy metabolism, DNA repair, and immune function. The normal functioning of these processes is crucial for maintaining overall health and delaying aging. Ames [35]’ research further established the important role of vitamins and minerals in DNA stability and repair. In summary, these research findings reveal the potential link between OBS and PhenoAgeAccel, supporting the crucial role of oxidative balance in the biology of aging and providing a theoretical basis for exploring innovative strategies to delay aging in the future.

In conclusion, the relationship between OBS and biological aging is multifactorial and complex, as oxidative stress, antioxidants, sex, high cholesterol levels, and lifestyle factors all play significant roles. The interactions and mechanisms of influence of these factors need further in-depth research and exploration to reveal the true relationship between OBS and aging and provide scientific evidence for the development of effective strategies to delay aging. We look forward to more research in the future to deepen our understanding of this field and explore the potential clinical applications of these findings.

This study, using a large dataset from NHANES spanning from 2001 to 2010 and employing rigorous scientific methods, delved into the relationship between OBS and PhenoAgeAccel, highlighting the crucial role of oxidative balance in slowing down the aging process. The research provides a scientific basis for formulating targeted health policies, intervention measures, and public health education, demonstrating its significant research advantages and practical application value. While this study has scientific value and practical significance, it also has some limitations: (1) Due to the cross-sectional design, the study mainly reveals associations between variables rather than causality. Although the study found a significant correlation between OBS and PhenoAgeAccel, it cannot determine whether this association arises from a causal relationship. Future research should use longitudinal designs or experimental methods to further explore the causal relationship between these variables. (2) The study relies on NHANES data from 2001 to 2010, which limits the timeliness and applicability of the study’s results. Over time, changes in population lifestyles, health status, and environmental factors may affect the relationship between OBS and PhenoAgeAccel. (3) Despite controlling for multiple variables, there may still have been unconsidered and uncontrolled confounding factors that could influence the actual relationship between OBS and PhenoAgeAccel. Factors such as genetics, environmental exposures, and life stress may also impact the aging process. (4) NHANES data are primarily based on the United States population, and the diversity of the United States population means that different racial, regional, and cultural backgrounds may lead to different physiological and health conditions. Therefore, the results of this study may not be directly extrapolated to populations in other countries and regions and need validation in different populations. (5) Some variables rely on self-reporting by participants (diet and exercise), which could introduce measurement errors and reporting bias, potentially affecting the accuracy of the study’s results.

This study has revealed a potential association between OBS and PhenoAgeAccel, indicating a significant correlation between higher OBS and lower PhenoAgeAccel. This finding suggests that optimizing oxidative balance, especially through dietary and lifestyle adjustments, may slow the aging trajectory. Furthermore, the study emphasizes the importance of OBS in assessing and understanding the aging process and provides a potential intervention point to slow down the aging process. Future research should further explore the biological mechanisms of this relationship and its applicability in different populations.

## Figures and Tables

**Figure f3-epih-46-e2024023:**
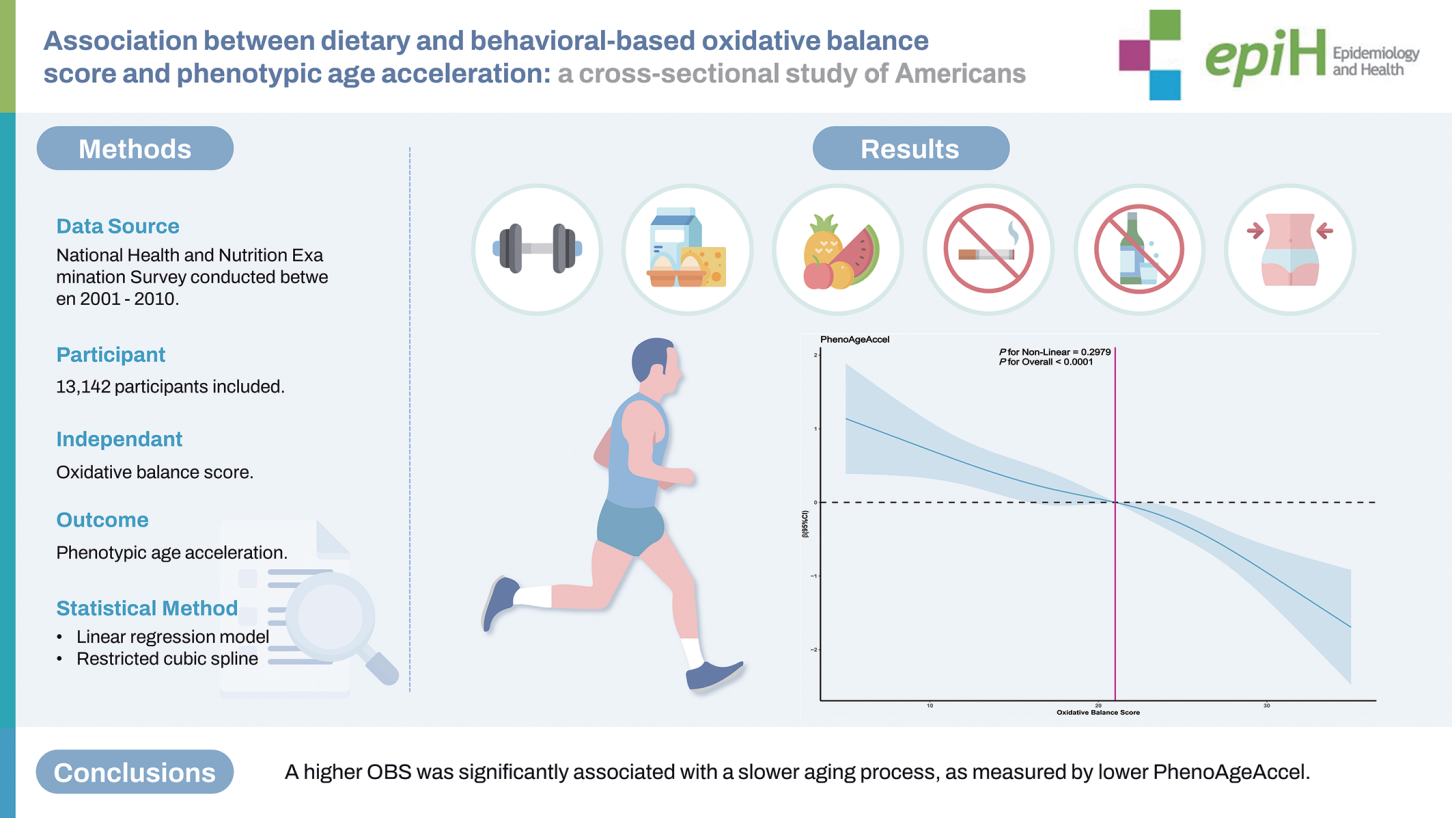


**Table 1.** Baseline characteristics of the study population

Characteristics	OBS^1^					
	Overall	Quartile 1	Quartile 2	Quartile 3	Quartile 4	p-value^2^
Total (n)	13,142	3,616	3,459	3,008	3,059	
PhenoAgeAccel	-4.86±0.08	-3.57±0.14	-4.51±0.13	-5.08±0.12	-6.20±0.15	<0.001
Phenotypic age	40.67±0.30	41.80±0.37	41.20±0.38	40.21±0.34	39.52±0.50	<0.001
Fasting glucose (mmol/L)	5.29±0.02	5.36±0.03	5.29±0.03	5.30±0.03	5.22±0.03	0.020
Alkaline phosphatase (U/L)	66.52±0.29	70.08±0.56	67.27±0.42	65.43±0.42	63.52±0.45	<0.001
Albumin (g/L)	42.94±0.05	42.55±0.08	42.85±0.08	43.02±0.07	43.31±0.08	<0.001
Creatinine (μmol/L)	79.52±0.29	81.09±0.60	79.97±0.56	78.78±0.40	78.32±0.41	<0.001
C-reactive protein (mg/dL)	0.37±0.01	0.45±0.02	0.38±0.01	0.37±0.01	0.27±0.01	<0.001
White blood cell count (1,000 cells/μL)	7.18±0.03	7.43±0.05	7.34±0.04	7.14±0.05	6.82±0.04	<0.001
Lymphocyte percent (%)	30.20±0.11	30.20±0.18	30.02±0.18	30.35±0.14	30.24±0.17	0.450
Mean cell volume (fL)	89.93±0.11	89.72±0.13	89.95±0.13	89.81±0.16	90.23±0.15	0.010
Red cell distribution width (%)	12.62±0.01	12.74±0.02	12.64±0.02	12.60±0.02	12.52±0.02	<0.001
Sex						0.350
Female	6,179 (48.75)	1,590 (47.18)	1,668 (49.40)	1,458 (49.81)	1,463 (48.59)	
Male	6,963 (51.25)	2,026 (52.82)	1,791 (50.60)	1,550 (50.19)	1,596 (51.41)	
Age (yr)						0.040
20-29	2,266 (18.58)	657 (20.94)	581 (18.26)	514 (18.30)	514 (16.97)	
30-39	2,329 (19.71)	555 (18.07)	576 (19.18)	582 (20.78)	616 (20.74)	
40-49	2,493 (22.81)	645 (22.04)	687 (23.66)	586 (22.83)	575 (22.65)	
50-59	2,064 (19.22)	547 (18.26)	535 (18.52)	481 (19.31)	501 (20.70)	
≥60	3,990 (19.70)	1,212 (20.70)	1,080 (20.39)	845 (18.78)	853 (18.93)	
Race						<0.001
Non-Hispanic Black	2,232 (8.66)	882 (13.80)	610 (9.24)	428 (7.20)	312 (4.67)	
Mexican American	2,218 (6.46)	607 (6.87)	592 (6.59)	539 (6.91)	480 (5.55)	
Non-Hispanic White	7,442 (76.53)	1,792 (70.33)	1,907 (75.02)	1,752 (77.64)	1,991 (82.70)	
Other race (including multi-racial and other Hispanic)	1,250 (8.35)	335 (9.00)	350 (9.14)	289 (8.25)	276 (7.08)	
Body mass index (kg/m^2^)						<0.001
<25.0	4,060 (33.35)	878 (25.71)	970 (29.98)	940 (33.12)	1,272 (43.91)	
25.0-29.9	4,628 (34.46)	1,261 (34.30)	1,242 (35.55)	1,082 (35.39)	1,043 (32.67)	
≥30.0	4,454 (32.19)	1,477 (39.99)	1,247 (34.48)	986 (31.49)	744 (23.41)	
Marital status						<0.001
Married/living with partner	8,437 (67.80)	2,189 (64.21)	2,238 (67.22)	1,974 (69.07)	2,036 (70.50)	
Never married	2,151 (16.31)	655 (18.10)	529 (16.18)	479 (15.83)	488 (15.25)	
Widowed/Divorced/Separated	2,554 (15.89)	772 (17.69)	692 (16.60)	555 (15.10)	535 (14.25)	
Education level						<0.001
Below	2,910 (13.72)	1,071 (19.34)	813 (14.78)	572 (12.29)	454 (8.83)	
High school	3,112 (23.95)	982 (29.68)	844 (24.96)	698 (23.66)	588 (17.95)	
Above	7,120 (62.33)	1,563 (50.99)	1,802 (60.26)	1,738 (64.05)	2,017 (73.22)	
Poverty to income ratio						<0.001
<1.30	3,242 (16.25)	1,159 (23.81)	844 (15.88)	666 (14.38)	573 (11.37)	
1.30-3.49	4,967 (34.83)	1,431 (38.03)	1,390 (37.92)	1,087 (32.81)	1,059 (30.68)	
≥3.50	4,933 (48.92)	1,026 (38.17)	1,225 (46.19)	1,255 (52.81)	1,427 (57.95)	
Smoking status						<0.001
Former smoker	3,488 (25.53)	914 (22.53)	924 (25.53)	823 (27.36)	827 (26.62)	
Non-smoker	6,766 (52.15)	1,566 (42.81)	1,738 (50.52)	1,621 (53.46)	1,841 (61.15)	
Current smoker	2,888 (22.32)	1,136 (34.65)	797 (23.94)	564 (19.18)	391 (12.22)	
Alcohol drinking status						<0.001
Former	2,369 (15.14)	797 (19.50)	631 (15.08)	485 (13.10)	456 (13.05)	
Never	1,502 (9.79)	450 (10.63)	400 (9.74)	319 (9.60)	333 (9.25)	
Mild	4,536 (36.84)	1,055 (29.71)	1,156 (35.39)	1,052 (37.20)	1,273 (44.50)	
Moderate	2,064 (17.28)	541 (16.68)	531 (16.85)	499 (18.20)	493 (17.41)	
Heavy	2,671 (20.95)	773 (23.48)	741 (22.94)	653 (21.90)	504 (15.79)	
Diabetes						<0.001
No	11,406 (90.51)	3,016 (88.72)	2,995 (89.97)	2,635 (90.75)	2,760 (92.48)	
Yes	1,736 (9.49)	600 (11.28)	464 (10.03)	373 (9.25)	299 (7.52)	
Hypertension						<0.001
No	7,947 (65.85)	1,980 (61.90)	2,038 (63.26)	1,889 (67.31)	2,040 (70.70)	
Yes	5,195 (34.15)	1,636 (38.10)	1,421 (36.74)	1,119 (32.69)	1,019 (29.30)	
Hyperlipidemia						<0.001
No	3,586 (28.61)	888 (24.88)	883 (27.03)	823 (28.35)	992 (33.82)	
Yes	9,556 (71.39)	2,728 (75.12)	2,576 (72.97)	2,185 (71.65)	2,067 (66.18)	

Values are presented as mean±standard error or number (weighted %). OBS, oxidative balance score; PhenoAgeAccel, phenotypic age acceleration.

1 OBS quartile ranges: Quartile 1=3 to 15; Quartile 2=16 to 21; Quartile 3=22 to 26; Quartile 4: 27 to 37.

2 One-way analysis of variance was applied to continuous variables, and the chi-square test was applied to categorical variables.

**Table 2.** Interaction effect tests

Variables	Q1	Q2	p-value	Q3	p-value	Q4	p-value	p for trend	p for interaction
Sex									<0.05
Male	Reference	-0.49 (-0.95, -0.04)	0.040	-0.88 (-1.28, -0.48)	<0.001	-1.27 (-1.81, -0.73)	<0.001	0.07	
Female	Reference	0.16 (-0.32, 0.63)	0.510	0.16 (-0.35, 0.66)	0.540	-0.33 (-0.83, 0.17)	0.190	<0.05	
Age (yr)									0.10
20-29	Reference	0.12 (-0.44, 0.68)	0.680	-0.16 (-0.76, 0.43)	0.590	-0.28 (-0.90, 0.34)	0.370	0.38	
30-39	Reference	0.01 (-0.72, 0.73)	0.980	-0.10 (-0.74, 0.54)	0.750	-0.56 (-1.24, 0.12)	0.100	0.57	
40-49	Reference	-0.32 (-1.16, 0.51)	0.440	-0.38 (-1.23, 0.47)	0.370	-0.75 (-1.56, 0.07)	0.070	0.46	
50-59	Reference	-0.39 (-1.09, 0.31)	0.270	-0.04 (-0.75, 0.67)	0.910	-0.46 (-1.37, 0.45)	0.320	0.36	
≥60	Reference	-0.40 (-1.11, 0.32)	0.270	-1.41 (-2.17, -0.65)	<0.001	-1.99 (-2.64, -1.34)	<0.001	<0.01	
Race									0.41
Non-Hispanic Black	Reference	-0.49 (-1.70, 0.73)	0.420	0.07 (-1.09, 1.24)	0.900	-1.23 (-2.24, -0.22)	0.020	0.49	
Mexican American	Reference	-1.14 (-1.77,-0.51)	<0.001	-0.89 (-1.69, -0.10)	0.030	-1.37 (-2.08, -0.66)	<0.001	0.21	
Non-Hispanic White	Reference	-0.01 (-0.46, 0.44)	0.960	-0.35 (-0.76, 0.05)	0.090	-0.73 (-1.14, -0.33)	<0.001	0.40	
Other race	Reference	-1.24 (-2.37,-0.11)	0.030	-1.02 (-2.20, 0.17)	0.090	-1.75 (-3.11, -0.40)	0.010	0.20	
Education level									0.51
Below	Reference	-0.24 (-1.07, 0.58)	0.560	-0.06 (-0.99, 0.87)	0.900	-1.38 (-2.32, -0.44)	0.005	0.05	
High school	Reference	0.01 (-0.71, 0.73)	0.980	-0.52 (-1.21, 0.18)	0.140	-0.67 (-1.40, 0.05)	0.070	0.95	
Above	Reference	-0.41 (-0.85, 0.03)	0.070	-0.55 (-0.98, -0.11)	0.020	-0.97 (-1.41, -0.52)	<0.001	0.58	
Poverty to income ratio									0.07
<1.30	Reference	-0.56 (-1.46, 0.33)	0.210	0.10 (-0.72, 0.92)	0.800	-1.12 (-1.80, -0.45)	0.001	0.49	
1.30-3.49	Reference	0.09 (-0.52, 0.71)	0.770	-0.52 (-1.03, -0.01)	0.040	-1.07 (-1.56, -0.57)	<0.001	0.22	
≥3.50	Reference	-0.42 (-0.86, 0.03)	0.070	-0.56 (-1.03, -0.10)	0.020	-0.80 (-1.29, -0.32)	0.002	0.47	
Body mass index (kg/m^2^)									0.77
<25.0	Reference	0.10 (-0.54, 0.74)	0.760	-0.39 (-1.09, 0.31)	0.270	-0.56 (-1.17, 0.04)	0.070	0.31	
25.0-29.9	Reference	-0.48 (-0.99, 0.04)	0.070	-0.53 (-1.05, -0.02)	0.040	-1.13 (-1.75, -0.52)	<0.001	0.10	
≥30.0	Reference	-0.32 (-0.92, 0.28)	0.290	-0.40 (-0.96, 0.15)	0.150	-1.18 (-1.82, -0.53)	<0.001	0.06	
Marital status									0.73
Married/living with partner	Reference	-0.24 (-0.64, 0.15)	0.220	-0.38 (-0.80, 0.03)	0.070	-0.87 (-1.30, -0.44)	<0.001	<0.05	
Never married	Reference	0.20 (-0.64, 1.05)	0.630	-0.17 (-0.98, 0.64)	0.670	-0.37 (-1.20, 0.46)	0.370	0.43	
Widowed/Divorced/Separated	Reference	-0.50 (-1.38, 0.37)	0.260	-0.94 (-1.82, -0.06)	0.040	-1.58 (-2.50, -0.66)	0.001	0.14	
Smoking status									0.90
Former smoker	Reference	-0.30 (-0.99, 0.39)	0.380	-0.36 (-1.17, 0.46)	0.380	-0.87 (-1.64, -0.11)	0.030	0.66	
Non-smoker	Reference	-0.35 (-0.75, 0.05)	0.080	-0.64 (-1.05, -0.23)	0.003	-1.01 (-1.50, -0.52)	<0.001	0.17	
Current smoker	Reference	-0.01 (-0.61, 0.59)	0.980	-0.03 (-0.57, 0.52)	0.920	-0.71 (-1.29, -0.13)	0.020	<0.05	
Alcohol drinking status									0.25
Former	Reference	-0.43 (-1.49, 0.63)	0.420	-0.86 (-1.76, 0.04)	0.060	-1.90 (-2.74, -1.06)	<0.001	0.05	
Never	Reference	-0.23 (-1.25, 0.78)	0.640	-0.44 (-1.55, 0.68)	0.440	0.17 (-1.10, 1.44)	0.790	0.59	
Mild	Reference	-0.35 (-0.95, 0.24)	0.240	-0.49 (-1.09, 0.12)	0.110	-1.12 (-1.68, -0.56)	<0.001	<0.01	
Moderate	Reference	-0.08 (-0.80, 0.65)	0.840	-0.30 (-1.13, 0.53)	0.470	-0.59 (-1.46, 0.28)	0.180	0.30	
Heavy	Reference	-0.07 (-0.69, 0.55)	0.830	-0.13 (-0.70, 0.43)	0.640	-0.46 (-1.22, 0.31)	0.230	0.38	
Diabetes									0.18
No	Reference	-0.09 (-0.42, 0.23)	0.560	-0.36 (-0.71, 0.00)	0.050	-0.74 (-1.08, -0.41)	<0.001	<0.01	
Yes	Reference	-1.43 (-2.91, 0.06)	0.060	-1.16 (-2.82, 0.51)	0.170	-2.53 (-4.48, -0.57)	0.010	0.65	
Hypertension									0.14
No	Reference	-0.42 (-0.84, 0.00)	0.050	-0.62 (-1.01, -0.22)	0.003	-1.10 (-1.52, -0.67)	<0.001	0.04	
Yes	Reference	0.33 (-0.29, 0.95)	0.290	0.08 (-0.51, 0.68)	0.780	-0.34 (-0.96, 0.28)	0.270	0.05	
Hyperlipidemia									0.02
No	Reference	-0.33 (-0.70, 0.03)	0.070	-0.15 (-0.57, 0.28)	0.490	-0.73 (-1.14, -0.33)	<0.001	0.07	
Yes	Reference	0.01 (-0.68, 0.70)	0.980	-0.93 (-1.68, -0.17)	0.020	-1.19 (-1.98, -0.41)	0.004	0.44	

Values are presented as Beta (95% confidence interval).

## Supplementary materials

Supplementary Material 1. Oxidative balance score (OBS)
