# Supplementary material for: Association between dietary and behavioral-based oxidative balance score and phenotypic age acceleration: a cross-sectional study of Americans
Source: Epidemiol Health. 2024 Jan 18;46:e2024023. doi: 10.4178/epih.e2024023 (PMC11176717; doi:10.4178/epih.e2024023)
Supplement: Supplementary Material 1. — Oxidative balance score (OBS) [file epih-46-e2024023-Supplementary-1.docx]

Supplementary Material 1. Oxidative balance score (OBS)

| Characteristic | Overall | Female | Male | Characteristic | Overall | Female | Male |
| --- | --- | --- | --- | --- | --- | --- | --- |
| Dietary fiber, g | 16.84(0.16) | 15.07(0.15) | 18.52(0.20) | Score Dietary Fiber, g | 1.09(0.01) | 1.10(0.02) | 1.09(0.02) |
| Total fat, g | 84.15(0.51) | 69.67(0.46) | 97.93(0.71) | Score Carotene (Retinol Equivalent), RE | 0.67(0.01) | 0.67(0.01) | 0.68(0.01) |
| Alpha carotene, mcg | 425.79(11.69) | 418.97(15.38) | 432.27(14.54) | Score Riboflavin, mg | 1.17(0.01) | 1.17(0.01) | 1.16(0.01) |
| Beta carotene, mcg | 2193.36(43.20) | 2228.45(56.12) | 2159.99(52.96) | Score Niacin, mg | 1.19(0.01) | 1.16(0.01) | 1.22(0.01) |
| Riboflavin, mg | 2.31(0.02) | 1.98(0.02) | 2.63(0.02) | Score Vitamin B6, mg | 1.15(0.01) | 1.14(0.02) | 1.16(0.02) |
| Niacin, mg | 25.79(0.16) | 20.99(0.17) | 30.36(0.22) | Score Total Folate, mcg | 1.12(0.01) | 1.09(0.02) | 1.15(0.02) |
| Vitamin B6, mg | 2.07(0.02) | 1.72(0.02) | 2.41(0.02) | Score Vitamin B12, mcg | 1.20(0.01) | 1.21(0.01) | 1.19(0.01) |
| Total folate, mcg | 428.71(3.59) | 370.79(3.83) | 483.81(4.68) | Score Vitamin C, mg | 0.99(0.01) | 0.99(0.02) | 0.99(0.01) |
| Vitamin B12, mcg | 5.72(0.08) | 4.59(0.07) | 6.78(0.11) | Score Vitamin E (α-tocopherol equivalent), mg | 1.01(0.01) | 1.02(0.01) | 1.00(0.01) |
| Vitamin C, mg | 89.95(1.32) | 82.35(1.39) | 97.17(1.72) | Score Calcium, mg | 1.19(0.01) | 1.21(0.02) | 1.18(0.01) |
| Vitamin E (α-tocopherol equivalent), mg | 7.79(0.07) | 6.95(0.08) | 8.59(0.08) | Score Magnesium, mg | 1.10(0.01) | 1.12(0.02) | 1.09(0.02) |
| Calcium, mg | 961.87(8.07) | 846.78(8.87) | 1071.35(9.84) | Score Zinc, mg | 1.14(0.01) | 1.14(0.01) | 1.14(0.01) |
| Magnesium, mg | 303.76(2.21) | 264.04(2.46) | 341.53(2.56) | Score Copper, mg | 1.09(0.01) | 1.08(0.01) | 1.10(0.01) |
| Iron, mg | 16.35(0.11) | 13.80(0.11) | 18.78(0.15) | Score Selenium, mcg | 1.12(0.01) | 1.11(0.01) | 1.13(0.01) |
| Zinc, mg | 12.71(0.11) | 10.33(0.09) | 14.96(0.17) | Score Total Fat, g | 0.93(0.01) | 0.93(0.01) | 0.92(0.01) |
| Copper, mg | 1.39(0.01) | 1.20(0.01) | 1.57(0.02) | Score Iron, mg | 0.88(0.01) | 0.89(0.01) | 0.87(0.01) |
| Selenium, mcg | 114.54(0.78) | 94.08(0.69) | 133.99(1.04) | Score Total Physical Activity MET (Metabolic Equivalent) | 1.13(0.01) | 1.15(0.02) | 1.12(0.02) |
| Alcohol, g | 10.79(0.37) | 6.21(0.31) | 15.14(0.53) | Score Alcohol, g | 0.84(0.01) | 0.85(0.01) | 0.82(0.01) |
| BMI (Body Mass Index), kg/m² | 28.21(0.10) | 28.02(0.14) | 28.40(0.10) | Score BMI (Body Mass Index), kg/m² | 0.93(0.01) | 0.93(0.02) | 0.94(0.02) |
| Total Physical Activity MET (Metabolic Equivalent) | 2389.70(80.68) | 1837.28( 66.69) | 2915.17(115.89) | Score Cotinine, ng/mL | 1.08(0.02) | 1.15(0.02) | 1.00(0.02) |
| Cotinine, ng/mL | 59.75(2.28) | 46.50(1.95) | 72.35(3.32) | OBS Dietary | 17.05(0.12) | 17.05(0.15) | 17.06(0.13) |
| Carotene  (Retinol Equivalent), RE | 200.52(3.99) | 203.16(5.21) | 198.01(4.89) | OBS Dietary Count | 16.00(0.00) | 16.00(0.00) | 16.00(0.00) |
|  |  |  |  | OBS Lifestyle | 3.98(0.03) | 4.09(0.03) | 3.88(0.03) |
|  |  |  |  | OBS Lifestyle Count | 4.00(0.00) | 4.00(0.00) | 4.00(0.00) |
|  |  |  |  | OBS | 21.03(0.14) | 21.13(0.17) | 20.93(0.15) |
|  |  |  |  | OBS Count | 20.00(0.00) | 20.00(0.00) | 20.00(0.00) |
